# Contributing factors of pregnant women’s beliefs towards mode of delivery: a cross-sectional study from Iran

**DOI:** 10.1186/s40748-018-0077-1

**Published:** 2018-05-02

**Authors:** Fereshteh Zamani-Alavijeh, Marzieh Araban, Akbar Hassanzadeh, Khadije Makhouli

**Affiliations:** 1 Department of Health Education and Promotion, School of Health, Isfahan University of Medical Sciences, Isfahan, Iran; 2 Social Determinants of Health Research Center, Ahvaz Jundishapur University of Medical Sciences, Ahvaz, Iran; 3 Department of Health Education and Promotion, Public Health School, Ahvaz Jundishapur University of Medical Sciences Campus, Ahvaz, Iran; 4 Department of Biostatistics and Epidemiology, School of Health, Isfahan University of Medical Sciences, Isfahan, Iran; 5 Student Research Committee, School of Health, Isfahan University of Medical Sciences, Hezarjarib Street, Isfahan, Iran

**Keywords:** Normal Vaginal Delivery, Mode of delivery, Attitude, Self-efficacy, Behavioral beliefs, Social support

## Abstract

**Background:**

Delivery is a critical event in every woman’s life. Under some medical conditions, women sometimes undergo a cesarean section to save the life of themselves and their infant. Understanding the factors associated with choosing the type of delivery leads to more effective health interventions and the promotion of maternal and newborn health. The aim of this study is to determine contributing factors in pregnant women’s beliefs toward the mode of delivery in a sample of women referring to Hojatieh Hospital in Isfahan, Iran.

**Methods:**

This cross-sectional study was conducted in September 2016 on 200 pregnant women (gestational age 30–37 weeks) referred to Hojatieh Hospital in Isfahan, Iran. The inclusion criteria of this study included healthy pregnancy and having no known cases (heart, pulmonary, and renal disease) and pregnancy complications (spotting, bleeding, headache, blurred vision), and willingness of pregnant women to complete the questionnaire. Data were collected through an eight-part questionnaire consisting of demographic factors, delivery intention, knowledge, perceived self-efficacy, attitude, perceived social, and private support in the presence of the researcher and the outcome of delivery were asked 2 weeks after the due date of delivery during a telephone call. Data were analyzed using SPSS16 software, independent t-test, chi-square, and logistic regression.

**Results:**

There was no significant differences between the mean scores of knowledge and private social support among individuals with two types of delivery (*P* > 0.05), but the mean scores of perceived self-efficacy and public social support in women who gave birth normal were significantly higher than those of women who gave birth by cesarean section (*P* < 0.05).

**Conclusion:**

The findings of this study showed that attitude and perceived self-efficacy were the most important predictors of delivery mode. Therefore, education based on the items included in these factors might be useful for choosing the delivery mode. The results might be used in evidence based midwifery practices in low and middle income countries to promote normal delivery and perhaps maternal health index.

## Background

Delivery is a beautiful event in women’s lives [1]. However, it could be stressful due to medical situations in which women undergo cesarean to save mothers or infants life [2]. Cesarean section (C-section) is the most common abdominal surgery [3], where abdominal wall muscles and uterus are cut [4]. fetal distress, high maternal age at delivery, delivery arrest, fetal distress, and abnormal fetal positions are common reasons for C-section [5]. Post C-section wound infection, pelvic infection, lung infection, pulmonary embolism, and anesthesia morbidities are associated with C-section [4]. It has been documented that economic and psycho-social costs of C-section are extremely higher than those of normal delivery [6]. Normal delivery could be the natural mode of deliveries in more than 90% of deliveries [7, 8].

International Federation of Obstetrics and gynecologists emphasized that conducting C-section without a medical reason is not moral [9]. World health organization (WHO) recommended no more than 10–15% of pregnancies by C-section [10]. According to the recommendations of Ministry of Health of Iran, the rate of C-section should be decreased to 20 and 25% in public and private hospitals, respectively [5]. The rate of C-section has been increased globally [11] such that in Canada, England, Scotland, Sweden and the United Arab Emirate it is 20, 25, 10, 14.2, and 10%, respectively [2]. During the recent years, the rate of C-section in Iran has been raised up in both private and public centers so that the country has been among the first four countries with high rates of C-section in the word [12]. Here, in Iran, 47% of deliveries are terminated by C-section [13], of which 40% are being done at maternal request [2]. C-section upon maternal request is among the common reasons for C-section in the world [14]. The reasons for C-section in Iran included but not limited to low facilities for overcoming maternal life-threatening risks, maternal request for tubal ligation, and considering C-section as a new method for delivery [15]. Additionally, conditions of normal delivery in hospital, doctors’ motivation, and social changes are accounted for C-section reasons in Iran [16]. Currently, C-section is being considered as a luxury mode for delivery in some regions [4]. Leon et al. claimed that economic and socio-cultural causes are reasons for C-section in developing countries. They further showed that fear of labor pain, vaginal examination stress, and low levels of knowledge are the other reasons for C-section [5].

Murthy et al. reported that doctors’ fear of legal punishment due to the poor prognosis of Normal Vaginal Delivery (NVD) is a reason for increasing C-section rate in USA [12]. Assadi et al. reported several complicated reasons contributed to high rates of C-section. In other words, the process of C-section decision-making, which is made by both pregnant women and doctors, is influenced by several factors [14]. Pregnant women’s self-efficacy, a false impression of the benefits of C-section, exaggerating the risks of vaginal delivery affect the mode of delivery [17]. Also, a positive correlation had been reported between attitude positive attitude toward C-section and requesting a C-section [18], perceived benefits and barriers, subjective norms are among other factors contributing to C-section [19]. Considering the fact that conducting C-section without medical reason has no moral logic and the high prevalence rate of C-section, the current study was conducted to identify the factors explaining the mode of delivery in Iran. Identifying beliefs toward a mode of delivery may help to reduce rates of maternal mortality and morbidity among low and middle-income counties.

## Methods

This cross-sectional study was carried out in September 2016. A total of 200 pregnant women (gestational age 30–37 weeks) from women’s clinic of Private Hojatieh Hospital in Isfahan, Iran, were selected by convenience sampling method.

Inclusion criteria were healthy pregnancy without any known heart, pulmonary and kidney diseases, no history of cesarean section for medical reasons, no complication during pregnancy, spotting, bleeding, headache, and blurred vision as well as tendency to participate in this study. Data were collected through the interviews performed by an educated research nurse using an eight-part questionnaire including demographic factors, delivery intention, knowledge, attitude, self-efficacy, general and specific perceived social support, and outcome of childbirth. This was asked by phone call 2 weeks after the due date of delivery. The present questionnaire was derived from relevant literature; e.g., Fathian [20], Zamani-Alavijeh [17], and Khaledi questionnaires [21]. Knowledge, attitude, and intention consisted of 10 items, 21 items, and 1 item, respectively, that have been from Fithian’s questionnaire. Perceived self-efficacy included 12 items from Zamani-Alavijeh’s questionnaire and general perceived social support from Khaledi’s questionnaire. According to the researcher’s team opinion, a question was added to the perceived social support questionnaire question. Validity and reliability of the questionnaires were approved by the mentioned researchers and Alpha Cronbach’s coefficient to be respectively 0.96, 0.87, 0.70. The Alpha Cronbach’s coefficient was recalculated for each construct by researcher where knowledge, attitude, perceived self-efficacy, and perceived social support constructs were 0.64, 0.92, 0.85, and 0.95, respectively. The first part of demographic factors includes 27 questions related to personal, social and pregnancy information. The second part includes one question related to the intention of pregnant for mode of delivery that has four possible options; i.e., probably normal delivery, probably cesarean, certainly normal delivery and certainly cesarean. The third part includes ten questions about knowledge that correct answer was awarded with 1 point and wrong answer was awarded as 0 and total score was between 0 and 100, with a higher score showing a better situation. A sample of the question about this construct is: What is the most common part of the body of the fetus that comes to the pelvis sooner from other parts? 1- Bottom of the fetus, 2- Head of the fetus, 3- Face of the fetus, and 4- Shoulder of the fetus. The fourth part includes 12 questions related to perceived self-efficacy that were assessed by the Likert criterion: I totally disagree, I disagree, No idea, I agree, I totally agree that were scored respectively as 0, 1, 2, 3, and 4. The questions that have a negative side were scored contrariwise and total score was between 0 and 100, with a higher score suggesting a better situation. A sample of the question about this construct is: I cannot stand the pain of normal delivery in any way. The fifth part includes 22 items related to attitude which were answered by the Likert criterion and options: I totally disagree, I disagree, No idea, I agree, I totally agree that were scored respectively as 0, 1, 2, 3, and 4, where the total score was between 0 and 100 with a higher score showing a better situation. The sample of the question about this construct is: Postpartum pain in cesarean is less than normal delivery. The sixth part includes 1 question related to private perceived social support that the score rating of question has been 7–1 and the total score has been between 6 and 0. The highest score was representative of a higher level of social support. The question about this construct is: In the process of childbirth, I have someone who provides me emotional support. The meaning of private social support is the presence of a trained individual along with the mother before giving birth and in the maternity ward. The seventh includes 12 questions related to general perceived social support that each question has a score between 1 and 7, with the total score being between 0 and 72. A higher score is the indicative of a higher level of social support. A sample of items is: When I need to, I always have a special person next to me. The meaning of general social support has been accompaniment and support of spouse, family, and friends during childbearing. The data were analyzed by SPSS 16 software, independent t-tests, Chi-square, and logistic regression.

## Results

A total of 200 pregnant women (gestational age of 37–30 weeks) without medical reasons for cesarean section with an average age of 29.1 years participated in the present study; 59% of the women were housekeepers and 5.78% of their husbands were self-employment; 71% of the women and 63.5% of their husbands had diplomas; of the current pregnancies, 74% were wanted of both husband and wife; 59% of people expressed that the most important reason for choosing a type of delivery in their newborn’s health. In the case of childbirth intention, in response to the question “If your doctor chooses to give you the type of delivery, what kind of childbirth do you select?” 17.1% selected “Probably vaginal delivery”, 32.7% selected “Probably cesarean” , 20.1% selected “Certainly vaginal delivery”, and 30.2% selected “Certainly cesarean”. Finally, 53% of them gave birth by cesarean section and 47% gave birth naturally. Table 1 presents the statistical indicators of different quantitative variables.

**Table 1:** The mean of quantitative variables

Variables	Mean	SD	Minimum	Maximum
Age (year)	29.1	5.02	15	44
Husband’s age (years)	31.3	5.6	18	47
Pregnancy number	1.7	0.8	1	5
Gestational age (week)	34.6	1.3	30	37
The number of family members	2.6	0.7	1	6

Independent t-test showed that there was no significant difference between a mean score of knowledge and specific social support among individuals with two types of delivery (*P* > 0.05). However, the mean of perceived self-efficacy score, attitude, and general social support in women who gave birth vaginally were significantly (*P* < 0.05) higher than those who gave birth by cesarean (Table 2).

**Table 2:** The mean score (from 100) of different constructs by type of delivery

Variables	Cesarean		Vaginal		*P*-value
	Mean	SD	Mean	SD	
Knowledge	30.4	19.5	33.3	18.9	0.28
Perceived self-efficacy	54.4	6.8	64.2	11.6	< 0.001
Attitude	43.4	8.3	55.7	8.2	< 0.001
Specific social support	87.2	9.9	89.3	9.2	0.12
General social support	83.1	14.9	87.6	13.2	0.02

Chi-square test showed a significant relationship between delivery type and delivery intention (*P* < 0.001). All who chose probably or certainly vaginal childbirth options before childbirth gave vaginal birth. Also, 70.8% of women who selected the option of probably cesarean and all those who definitely chose cesarean were given cesarean delivery.

Logistic regression analysis showed that perceived self-efficacy, attitudes, and general social support scores in 81.9% of the cases could accurately predict the delivery type. Among variables whose scores had a significant relationship with the type of delivery, attitude and perceived self-efficacy variables, in the order of their appearance, had the most ability to predict the type of delivery, and the social support variable in the presence of two other variables did not have much ability in this regard. The lambda coefficient between delivery intention and delivery type was equal to 0.796, indicating acceptable level of agreement (Table 3).

**Table 3:** Logistic regression analysis to predict the type of delivery according to the score of different structures

Variables	Beta	Wald	*P*-value	OR	95% C.I for OR	
					Lower Limit	Upper Limit
Perceived Self-Efficacy	0.078	9.40	0.002	1.08	1.03	1.14
Attitude	0.188	31.38	< 0.001	1.21	1.13	1.29
General Social Support	0.004	0.080	0. 78	1	0.98	1.03

## Discussion

The aim of this study was to assess factors contributing to select the mode of delivery in women referred to Hojjatieh hospital, Iran. The results slowed that self-efficacy, attitude, and general social support could account for 81.9% in the prediction of delivery mode. Among these factors, attitude and self-efficacy had a greater predictive power for selection of delivery mode. In line with our study, a study by Latifnezahed et al. revealed that motivation toward C-section is stronger than that of normal delivery [22]. In contrast, Hajian et al. showed that pregnant women’s especially primiparous fear of doctors nonattendance was the common reason of fear of NVD [23]. The present research showed that private social support social support was about 87.2% among women and the women stated that presence of doctors, midwife, or doula could lead to the reduction of fear of NVD. This study showed a positive attitude toward C-section, which in line with Movahed et al., showing a positive relationship C-section and attitude [18].

Although Forstholm et al. found that being worried about the health of mother and baby was the main reason for selecting C-section among primiparous women [24], the current research showed that being worry about baby’s health was the main reason for selecting C-section among women. This difference may result from the differences between women’s background variables.

False beliefs, normative beliefs, and not having enough knowledge were determinants of the method of delivery in an earlier research [25]. This result is not in line with our study in which the levels of knowledge were not different among women with C-section and NVD.

Tavassoli et al. reported positive correlations between selecting C-section and being aware of C-section morbidities, doctors’ motivation, fear of NVD, maintaining body beauty, and socio-economic class of women [26].

Mohammaditabar showed that private hospitals, age less than 21 years, and a positive attitude of medical care team toward C-section are associated with C-section [27]. The fear of NVD and self- efficacy for coping with fear are in line with the results of the current research.

Vafaee et al. showed that worry about baby health and an unfavorable change in genital tract and husband’s view were the mean causes of C-section [28]. Being worried about baby’s health and change in genital tract are in common reasons between the current research and those of Vafaee et al.

Although Sharqi et al. showed that doctor’s recommendation was the main reason for C-section, the current research showed that women themselves prefer C-section [29].

Constructs used in the present study could account for 85% prediction in delivery mode, while previous works have shown a lower variance.

## Limitation

The results of current research are not generalizable to all pregnant women.

## Conclusion

The findings of this study showed that attitude and perceived self-efficacy were the most important predictors of delivery mode. Therefore, education based on the items included in these factors might be useful for choosing the delivery mode. The results might be used in evidence based midwifery practices in low and middle income countries to promote normal delivery and perhaps maternal health index.

## Abbreviations

CVI: Content validity index
CVR: Content validity ratio
NVD: Normal Vaginal Delivery
